# The Role of Alarmins in the Pathogenesis of Rheumatoid Arthritis, Osteoarthritis, and Psoriasis

**DOI:** 10.3390/cimb46040228

**Published:** 2024-04-19

**Authors:** Kajetan Kiełbowski, Wiktoria Stańska, Estera Bakinowska, Marcin Rusiński, Andrzej Pawlik

**Affiliations:** 1 Department of Physiology, Pomeranian Medical University, 70-111 Szczecin, Poland; kajetan.kielbowski@onet.pl (K.K.); esterabakinowska@gmail.com (E.B.); rusinski.marcin92@gmail.com (M.R.); 2 Department of Medical Biology, Medical University of Warsaw, 00-575 Warsaw, Poland; wstanska@gmail.com

**Keywords:** alarmins, high-mobility group box 1, S100 proteins, rheumatoid arthritis, osteoarthritis, psoriasis

## Abstract

Alarmins are immune-activating factors released after cellular injury or death. By secreting alarmins, cells can interact with immune cells and induce a variety of inflammatory responses. The broad family of alarmins involves several members, such as high-mobility group box 1, S100 proteins, interleukin-33, and heat shock proteins, among others. Studies have found that the concentrations and expression profiles of alarmins are altered in immune-mediated diseases. Furthermore, they are involved in the pathogenesis of inflammatory conditions. The aim of this narrative review is to present the current evidence on the role of alarmins in rheumatoid arthritis, osteoarthritis, and psoriasis. We discuss their potential involvement in mechanisms underlying the progression of these diseases and whether they could become therapeutic targets. Moreover, we summarize the impact of pharmacological agents used in the treatment of these diseases on the expression of alarmins.

## 1. Introduction

Alarmins are considered danger-associated molecular patterns (DAMPs); they are endogenous immune-activating factors associated with cell injury and death. The main role of these molecules is to alert the immune system and initiate inflammatory responses [1]. Inflammatory mediators, such as tumor necrosis factor-α (TNF-α); interleukin (IL)-1β; IL-6; IL-8; and chemokine ligand (CC motif) 2 (CCL2), 3 (CCL3) and 4 (CCL4), as well as nitric oxide, are released by neutrophils and macrophages, the secretions of which are induced by alarmins [2,3,4,5]. Cells release alarmins in response to various stimuli, such as thermal, chemical, physical, metabolic, or infectious stimuli. Analysis of pathways stimulated by alarmins is fundamental to better understanding inflammatory processes, given that they may be biomarkers of immune-mediated diseases.

The pathogenesis of rheumatoid arthritis (RA) and inflammatory skin diseases, such as psoriasis, is associated with dysregulated immune responses and tissue damage. RA is a chronic inflammatory disease associated with multiple joint malfunctions. The pathogenesis of RA is complex and not fully understood; these mechanisms involve interactions between RA fibroblast-like synoviocytes (RA-FLSs) and immune cells, epigenetic factors, and the impact of environmental stimuli [6,7,8]. Similarly, the pathogenesis of psoriasis involves interactions between immune cells and keratinocytes. Abnormal inflammatory responses occurring in the skin lead to the excessive proliferation of keratinocytes, differentiation impairment, and the formation of pathological skin eruptions. Osteoarthritis (OA) is another disease with inflammatory components that play a role in its pathogenesis. It is a whole-joint disorder, and since it is associated with cartilage degeneration, alarmins may be involved in OA-related inflammatory mechanisms. The aim of the present review is to explore the current evidence on the role of alarmins in the pathogenesis of RA, OA, and psoriasis, with a focus on understanding their molecular mechanisms, involvement in disease progression, and potential therapeutic targets.

## 2. Materials and Methods

To perform this narrative review, we conducted a thorough literature search through the PubMed and Embase databases. We used the following keywords: ‘rheumatoid arthritis’, ‘osteoarthritis’, ‘psoriasis’, ‘high mobility group box 1’, ‘S100 proteins’, ‘interleukin-33’, ‘interleukin-1α’, ‘heat shock proteins’, ‘defensins’, cathelicidin’, ‘thymic stromal lymphopoietin’, and their combinations. We have discussed original articles that investigated the impact of alarmins on the pathogenesis of these diseases. Furthermore, we have analyzed studies that examined whether therapeutic agents used in the treatment of the abovementioned diseases affect the expression of alarmins.

## 3. Brief Overview of Alarmins

Alarmins represent a broad group of molecules, each with distinct structural and functional properties. Among the most common peptides mentioned are high-mobility group box 1 (HMGB1), S100 proteins, interleukin-33 (IL-33), interleukin-1α (IL-1α), heat shock proteins (HSPs), defensins, cathelicidin, thymic stromal lymphopoietin (TSLP), and others.

HMGB1, also known as amphoterin, is a non-histone chromatin-binding protein present in all cell types. The structure of human HMGB1 consists of 215 amino acid residues. They are arranged in two domains (HMG A box and HMG B box), a C-terminal acidic tail, and an N-terminal region [9]. Despite its subcellular location, HMGB1 exerts multiple functions. HMGB1 acts as a chaperone in the nucleus, maintaining chromosome structure and function. However, in the cytoplasm, by binding to the Beclin-1 (BECN1) protein, it can promote autophagy [9]. Extracellular HMBG1 can be passively released after cell death or actively secreted. It regulates inflammation and immune responses through various receptors or direct uptake, acting as DAMP. Many factors are responsible for regulating the secretion and release of HMGB1, such as post-translational modifications (e.g., acetylation, ADP-ribosylation, phosphorylation, and methylation) and the molecular mechanisms of cell death (e.g., apoptosis, pyroptosis, necroptosis, alcoptosis, and ferroptosis). The main receptors for HMGB1 are toll-like receptors (TLRs) and the receptor for advanced glycation endproducts (RAGE) [10]. Its potential role in immunological processes is exerted by the ability to activate nuclear factor kappa B (NF-kB) and subsequently stimulate the expression of IL-1 and TNF-α.

S100 proteins belong to a family of approximately 24 members. Each S100 subunit consists of four α-helices and two EF-hands (helix–loop–helix motifs), which are Ca^2+^-binding domains [11]. The term S100 is associated with their capacity to dissolve 100% of saturated ammonium sulfate solution [12]. The activation of S100 proteins consists of two mechanistic regulatory steps [13]. The initial step is to bind transition metals (Ca^2+^, Zn^2+^, Cu^2+^, and Mn^2+^) for folding [14,15]. The second step involves the synthesis of homo- and heterodimers [16]. After binding with Ca^2+^, the conformation changes, which allows the protein to exert its biological effects. The main functions of S100 proteins are regulations in cell apoptosis, proliferation, differentiation, gene expression, enzyme activation, inflammatory responses, and energy metabolism [17].

IL-1α is a pro-inflammatory cytokine that belongs to the IL-1 family. All 11 family members contain a single β-trefoil structure that binds to immunoglobulin-like receptors. IL-1α initiates an inflammatory process, but its isoform, IL-1β, propagates and maintains it. Both isoforms act via IL-1 receptors I and II (IL-1R-I and IL-1R-II). IL-1 plays an important role in responses to stress, regulating the expression of genes. Moreover, the IL-1 receptor antagonist (IL-1RA) is another molecule that binds to the IL-1 receptor. However, it inhibits inflammatory signals [18].

IL-33 is another member of the IL-1 family. Its structure is composed of two evolutionarily preserved domains: the N-terminal nuclear domain and the C-terminal IL-1-like cytokine domain. These domains are distinctly separated by a unique, divergent central segment [19]. It is expressed in the nuclei of keratinocytes, fibroblasts, vascular endothelial cells, dendritic cells, macrophages, and mast cells [20]. IL-33 mediates type 2 innate immune reactions and allergic inflammation. Furthermore, it acts as a chemokine, activating neutrophils and macrophages [21]. It exerts its functions by interacting with a range of target cells equipped with the suppression of the tumorigenicity 2 (ST2) receptor, such as mast cells and type 2 innate lymphoid cells [19]. Binding the IL-33 to ST2 leads to the transduction of inflammatory signals to Th2 cells, regulatory T cells, type 2 innate lymphocytes (ILC2), dendritic cells, macrophages, eosinophils, basophils, and mast cells. However, the soluble ST2 (sST2) receptor does not transduce the intracellular signals but rather acts as a decoy receptor, inhibiting IL-33 inflammatory signals. The transmembrane receptor for IL-33 has the ability to regulate genes by inducing the myeloid differentiation factor 88 (MyD88)-dependent activation of NF-kB and mitogen-activated protein kinase (MAPK).

TSLP belongs to the IL-2 cytokine family [22]. It is primarily produced by epithelial cells found in the gastrointestinal tract, lungs, and skin. Nevertheless, it is noteworthy that TSLP expression is also observable in various other cell types, including dendritic cells (DCs), basophils, and mast cells [23]. Various endogenous and environmental stimuli, including pro-inflammatory cytokines, tryptase, invading pathogens, allergens, irritants, pollutants, and cigarette smoke, can provoke epithelial cells at barrier surfaces to secrete TSLP.

## 4. Alarmins and Rheumatoid Arthritis

### 4.1. High-Mobility Group Box 1

Rheumatoid arthritis is an autoimmune disorder associated with joint swelling, as well as cartilage and bone damage. The pathogenesis of this disease involves numerous interactions between immune cells and FLSs that acquire an aggressive phenotype [24]. RA is a chronic disease with increasing prevalence [25], and both genetic (major histocompatibility complex alleles) and environmental (female sex, smoking, dust exposure, and diet) factors are considered risk factors [26]. The pathogenesis of RA is not entirely known, and thus, investigating the mechanisms associated with the development of RA may introduce novel treatment strategies. In this section, we will focus on the role of alarmins in this form of autoimmune arthritis.

To begin with, higher blood levels and the increased expression of HMGB1 in peripheral blood mononuclear cells have been detected in RA patients [27,28,29]. Furthermore, serum concentrations of HMGB1 are elevated in RA patients with an active disease [30]. Moreover, the level of HMGB1 will be greater in the synovial fluid of RA patients compared with those with OA [31]. Treatment of RA patients is associated with the reduced synovial expression of HMGB1 compared with untreated cohorts [32], thus suggesting that it could play a role in the pathogenesis of RA. Several studies have demonstrated various mechanisms involving a DNA-binding protein that could contribute to the disease’s progression. First, HMGB1 impacts the functionality and activity of RA-FLSs. For instance, it is associated with RA-FLS proliferation since siRNA-mediated silencing reduces synoviocyte proliferation. In addition, silencing HMGB1 reduces the migration and invasion of RA-FLSs and reduces MMP-13 and MMP-2 expression [32]. In another study, HMGB1 was found to synergize with lipopolysaccharide (LPS) to enhance RA-FLS proliferation and increase the expression of IL-6 and MMPs. Mechanistically, the combination of LPS with HMGB1 was found to stimulate the p38 and NF-*κ*B pathways [33].

The pathogenesis of RA involves angiogenesis, as the formation of new blood vessels stimulates the infiltration of immune cells and drives synovial inflammation [34]. Importantly, HMGB1 has been associated with the enhanced process of angiogenesis [35]. The molecule can be internalized by the endothelial cells through RAGE, which eventually promotes the release of vascular endothelial growth factor (VEGF) [36]. Accordingly, in RA animal models, exogenous HMGB1 stimulates the development of blood vessels, which are reduced when an HMGB1 inhibitor is introduced [37]. In another study, it was demonstrated that HMGB1 could stimulate RA-FLSs to upregulate hypoxia-inducible factor 1α (HIF-1α) through NF-κB [38]. Furthermore, HMGB1 enhances inflammatory responses that promote the progression of RA. Specifically, it activates immune cells and mediates their migration and secretion of inflammatory cytokines. Macrophages are one of the key cells contributing to the pathogenesis of RA, and they do so by secreting a number of pro-inflammatory cytokines, including tumor necrosis factor-alpha (TNF-α), a molecule targeted by several biological agents used in the treatment of RA. Moreover, macrophages can stimulate T cells, another major cellular subtype involved in the pathogenesis of inflammatory arthritis [39]. In an early study by Andersson et al., the stimulation of peripheral blood mononuclear cells (PBMCs) with HMGB1 enhanced the gene expression and protein secretion of TNF-α [4]. Similarly, stimulating macrophages derived from synovial fluid with HMGB1 increases the production of inflammatory mediators, TNF-α, interleukin-1 beta (IL-1β), and IL-6. The blockade of the RAGE receptor suppresses the enhanced secretion of TNF-α [31]. Additionally, HMGB1 is involved in the process of macrophage migration. Studies have demonstrated that chemokines, which attract immune cells toward inflamed synovium, are upregulated in patients with RA. For instance, high levels of C-X-C motif chemokine ligand 12 (CXCL12) are found in the synovial fluid of patients in the active stage of RA [40]. CXCL12 can create a complex with HMGB1 to further enhance its chemotactic properties. In a study by Cecchinato and colleagues, the authors demonstrated that, in the presence of CXCL12, even low concentrations of HMGB1 induced the migration of monocytes derived from RA patients with active disease. Mechanistically, the authors found that the JAK/STAT and COX2 pathways take part in monocyte migratory responses [41]. Another macrophage-related mechanism occurring in RA is pyroptosis, a programmed cell death associated with the release of IL-18 and IL-1β [42]. Several studies have demonstrated that HMGB1 enhances macrophage pyroptosis [43,44]. In addition, different HMGB1 redox isoforms induce various responses. Disulfide HMGB1 was found to enhance the pro-inflammatory phenotype similar to the classic M1 subtype [45]. The suppression of HMGB1 expression is associated with reduced inflammatory responses in macrophages. Specifically, the transfection of microRNA-25 (miR-25) suppresses the expression of HMGB1 and reduces the production of pro-inflammatory mediators [46].

**Figure 1:**
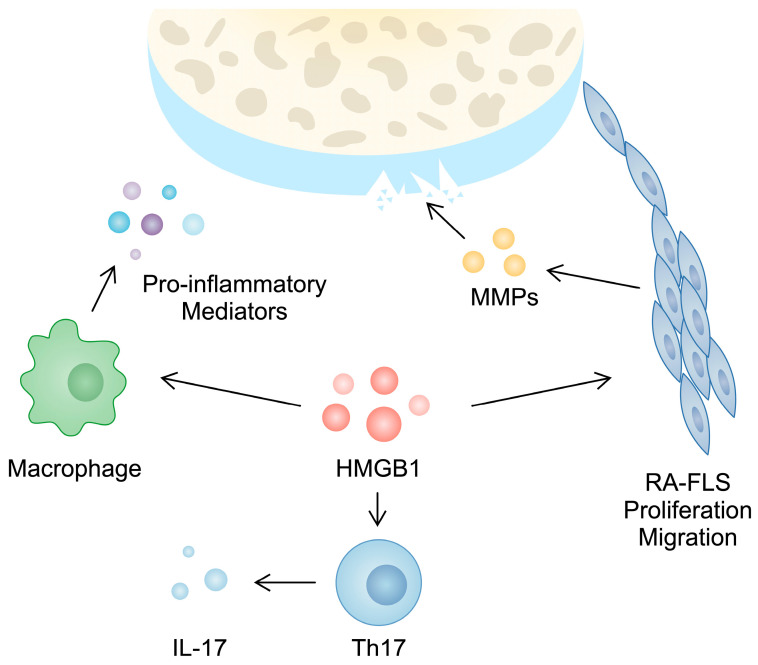
Hypothetical involvement of HMGB1 in the pathogenesis of rheumatoid arthritis. Firstly, it is involved in the invasive features of RA-FLSs since its silencing is associated with reduced migration, proliferation, and the secretion of matrix metalloproteinases. Secondly, stimulating macrophages and CD4+ T cells with HMGB1 are associated with the enhanced production of pro-inflammatory cytokines and Th17 markers, respectively.

Furthermore, HMGB1 takes part in the activity of T cells, which play a major role in the progression of RA. For instance, a higher population of Th17 cells can be observed in the PBMCs of RA patients compared with controls [47]. These cells produce several cytokines, including IL-17, which is responsible for inducing angiogenesis, synovial inflammation, and cartilage and bone damage [48]. In PBMCs from RA patients, a positive correlation between HMGB1 and IL-17 gene expression has been observed. Moreover, in an in vitro experiment, the treatment of CD4+ T cells with HMGB1 enhanced the presence of Th17 markers [28]. This finding was confirmed in another in vitro analysis performed by Su and collaborators [49]. Taken together, these studies broadly demonstrate the role of HMGB1 in the pathogenesis and inflammatory responses typical of RA (Figure 1).

Importantly, it is interesting to note the role of drugs in the expression of HMGB1. Firstly, HMGB1 gene polymorphisms are associated with RA treatment responses. In a study by Xu et al., the authors found that allele G of the rs2249825 polymorphism was associated with refraction susceptibility to the treatment with conventional synthetic disease-modifying antirheumatic drugs (csDMARDs) [50]. The synovial expression of HMGB1 decreased in patients treated with methotrexate (MTX), as compared with RA patients, who did not receive the antifolate [32]. MTX is broadly used in the treatment of RA, and few studies have investigated its effect on HMGB1. The treatment of monocytes with MTX in the presence of LPS/IFNγ does not change the secretion of TNF or HMGB1 [51]. However, when the RAGE receptor was stimulated with a truncated version of HMGB1 in a murine macrophage-like cell line, the introduction of MTX significantly reduced the secretion of TNF [52], indicating that MTX might affect HMGB1/RAGE interaction. In addition, HMGB1 is involved in the resistance of RA-FLSs to MTX. Specifically, MTX has been shown to enhance autophagy in RA-FLSs, a process that has been used to evade apoptosis. Moreover, the drug increases the expression of HMGB1 in RA-FLSs. Importantly, silencing HMGB1 significantly enhances apoptosis induced by MTX [53]. Recently, HMGB1 has been suggested to be involved in MTX-induced hepatotoxicity. The protein expression of alarmin is increased in the hepatic tissues of mice treated with MTX. *HMGB1*-knockdown experiments have demonstrated that this alarmin is involved in MTX-stimulated ferroptosis and autophagy [54].

Few studies have evaluated the associations between HMGB1 and other agents used in the treatment of RA. TNF (etanercept) and IL-1β (anakinara) inhibitors do not suppress the release of HMGB1 from monocytes [51]. Similarly, there are no significant differences in the protein and gene synovial expressions of HMGB1 in RA patients treated with infliximab, another monoclonal antibody-targeting TNF [55]. However, the serum level of HMGB1 significantly declines in RA patients treated with metformin [30], a biguanide derivative that was previously found to bind HMGB1 [56]. Taken together, HMGB1 seems to be involved in the pro-inflammatory mechanisms implicated in the pathogenesis of RA. Targeting HMGB1 could represent a future treatment strategy that needs to be further investigated.

### 4.2. S100 Proteins

S100 proteins represent another family of alarmins investigated in the context of RA. To begin with, many studies have evaluated the role and presence of calprotectin, a molecule composed of S100A8 and S100A9, in RA patients. In serum, significantly greater concentrations of calprotectin are detected in patients with RA compared with controls [57,58,59]. Thus, calprotectin has been evaluated as a diagnostic biomarker. The combination of serum calprotectin, anti-CCP3, and rheumatoid factor (RF) is associated with a promising positive predictive value for detecting RA occurrence within three years [60]. Furthermore, greater levels of calprotectin in the synovial fluid [61] and elevated expressions of S100A8 and S100A9 have been found in the monocytes of patients with RA [62]. Importantly, calprotectin levels are associated with disease activity, even in patients with low concentrations of CRP [63]. In a meta-analysis by Bae et al., circulating calprotectin was positively correlated with DAS28 and CRP [61]. Intriguingly, even a greater association with pro-inflammatory mediators was observed in ACPA-positive patients [64]. Using calprotectin as a marker of disease activity has been also suggested in patients in whom it would be difficult to measure inflammatory parameters due to the administration of tocilizumab [65]. Additionally, serum calprotectin concentrations could be used to identify patients with synovitis detected on power Doppler ultrasounds [66,67]. In line with these findings, serum levels of S100A8/A9 decrease in RA patients after three months of treatment, and lower levels have been detected in patients achieving remission [68].

Overall, multiple studies have demonstrated the potential role of calprotectin as a diagnostic biomarker, as well as a laboratory parameter, that could be used to monitor disease activity, even in patients with lower concentrations of other RA parameters, such as CRP. However, monitoring calprotectin to predict flares and relapses remains controversial. De Moel et al. showed that higher levels of circulating calprotectin at the moment of treatment tapering could indicate potential flares within twelve months [69]. By contrast, Romand and collaborators demonstrated that monitoring calprotectin levels does not assist in detecting RA relapses [70].

S100A8 and S100A9 impact the functionality of immune cells, including neutrophils and macrophages. Neutrophils play a significant role in the pathogenesis of RA since they release neutrophil extracellular traps (NETs), structures involved in the propagation of inflammation and a major source of citrullinated proteins [71]. Evidence suggests that S100A9/S100A9 can be released in NETs, indicating that it could be used as a marker of neutrophil-derived structures. Furthermore, it contributes to neutrophil activation [72]. The induction of pro-inflammatory cytokine secretion has been found to depend on the presence of phosphorylated S100A9 [73], and importantly, the phosphorylated protein is present in the synovial fluid of RA patients [74]. Calprotectin is also implicated in the activity of macrophages. The presence of S100A8/A9 has been found in macrophages from the synovial tissues of RA patients. Through the p38 and NF-κB pathways, S100A8/A9 can enhance the production of pro-inflammatory mediators in monocytes [75]. In addition, these alarmins stimulate NOD-, LRR-, and pyrin-domain-containing protein 3 (NLRP3) inflammasome, which is associated with inflammation and pyroptosis [76]. The stimulation of THP-1 macrophages with S100A8 enhances the protein expression of NLRP3 and its downstream elements, including GSDMD, IL-1β, and cleaved caspase-1, among others [77]. S100A8 and S100A9 are also implicated in other inflammatory pathways associated with T cells. The previously mentioned Th17 cells secrete IL-22 as well, which is implicated in the pathogenesis of RA [48]. In an in vitro experiment, the stimulation of RA-FLSs with IL-22 enhanced the mRNA expression of S100A8 and S100A9, which could then interact with immune cells to promote inflammation [78]. Additionally, these alarmins can be expressed by bone and cartilage cells [79]. In S100A9-/- antigen-induced arthritis, significantly reduced bone erosion has been observed. Moreover, the introduction of S100A8 promotes osteoclast formation during osteoclastogenesis [80]. By contrast, another study by Di Ceglie and colleagues suggested that the presence of S100A9 suppresses the differentiation of monocytes toward osteoclasts [81].

**Figure 2:**
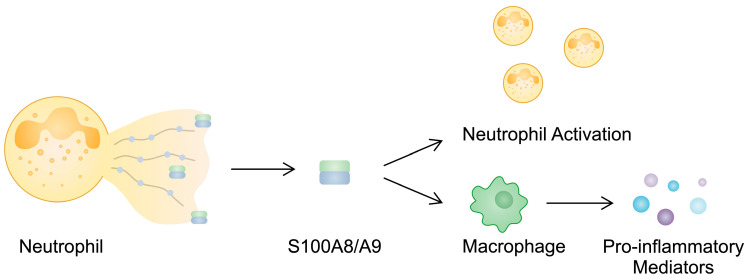
S100A8/A9 is released during NETosis, which then activates other neutrophils and enhances macrophages so that they secrete pro-inflammatory cytokines.

Taken together, S100A8 and S100A9 could play a role in RA progression (Figure 2). Importantly, the use of neutralizing antibody-targeting S100A9 results in decreased disease incidence and activity, as well as reduced cartilage and bone damage, in RA murine models [82]. In addition, the administration of the recombinant apoptosis inhibitor of macrophages (AIM) suppresses inflammatory responses in murine autoimmune arthritis models. Simultaneously, AIM has been suggested to reduce the expression of S100A9, but the difference is not significant (*p* = 0.065) [83]. These proteins could also serve as markers of responses. However, taking into account the relationship between S100A8/A9 and disease activity, it is intriguing that higher serum levels of S100A9 have been observed in responders to treatments with MTX ad etanercept [84]. Similarly, higher serum concentrations of calprotectin have been found in responders to treatments with adalimumab or etanercept [85].

The presence of other members of the S100 family has been studied as well. A greater expression of S100A11 (calgizzarin) has been found in the synovial tissue, and higher synovial fluid levels of S100A11 have been observed in patients with RA compared with OA [86]. Its levels positively correlate with disease activity and inflammatory markers [86,87], suggesting its involvement in disease progression. Similarly to the previously described proteins, S100A11 is associated with neutrophils. Specifically, the release of calgizzarin occurs during NETosis. In turn, the alarmin affects inflammation by enhancing IL-6 and TNF secretion in neutrophils derived from healthy patients. Intriguingly, S100A11 does not stimulate pro-inflammatory responses in neutrophils obtained from RA patients [87], suggesting that it might be involved in the early stages of RA pathogenesis or that RA cells exert some sort of tolerance. Another alarmin with elevated concentrations in the sera of RA patients is S100A12 [88]. Moreover, the levels of S100A12 are higher in the synovial fluids of RA patients compared with OA, where it was not detected [89]. Importantly, its concentrations are significantly correlated with ultrasonography scores, as well as clinical and inflammatory parameters [90]. Additionally, S100A12 stimulates the presence of neutrophils [89] and osteoclastogenesis [91], cell types highly implicated in RA progression.

### 4.3. Interleukin-33

Another alarmin studied in the context of RA is IL-33, a member of the IL-1 family. After being released from injured cells or tissues, an active and pro-inflammatory form of IL-33 acts through the suppression of the tumorigenicity 2 (ST2) receptor [92]. NF-κB, ERK, and MAPK are considered downstream elements of IL-33/ST2 signaling [93]. Several studies have investigated the involvement of IL-33 in RA. To begin with, different results have been obtained regarding the concentrations of this cytokine. In a study by Hong and collaborators, significantly higher serum levels of IL-33 were observed in RA patients compared with healthy controls. Furthermore, greater concentrations of IL-33 have been noted in the synovial fluids of RA patients when compared with patients with OA [94]. In line with these findings, Tang et al. also observed higher levels of IL-33 in RA synovial fluid, as compared with OA [95]. However, in another report by Talabot-Ayer and colleagues, the difference between the groups was not statistically significant. Furthermore, the authors demonstrated that the synovial expression of RA and OA does not differ significantly [96]. Importantly, positive correlations between serum and synovial fluid IL-33 levels with disease activity parameters, autoantibody concentrations, and inflammatory mediators have been found [94,95], which could suggest its involvement in the pathogenesis of the disease. Nevertheless, its involvement in mechanisms underlying the pathogenesis of the disease may be more complex. Recently, Poole and collaborators found an inverse correlation between IL-33 levels and the risk of RA-associated interstitial lung disease [97].

Firstly, the genetic polymorphism of *IL-33* has been associated with disease susceptibility. Specifically, the rs10975519 CC genotype has been suggested to decrease the susceptibility to developing RA among females [98]. Secondly, in the autoantibody-induced arthritis animal models, the introduction of IL-33 was associated with significantly greater clinical scores for arthritis [99]. IL-33 is involved in various molecular mechanisms involving RA-related cells. It impacts the functionality of RA-FLSs by regulating the expression of pro-inflammatory mediators. Nonetheless, mechanisms induced by IL-33 might depend on the cellular context, as Wu et al. demonstrated that various doses of IL-33 can differently induce the expression of pro-inflammatory mediators [100]. By contrast, in an in vitro experiment, Lee and collaborators showed that the overexpression of IL-33 in stimulated RA-FLSs reduces the activity of NF-κB and pro-inflammatory cytokines [101]. As previously mentioned, the ERK pathway is a downstream element of IL-33 signaling. Intriguingly, it also seems to take part in the regulation of IL-33 secretion. Specifically, miR-483-3p is a molecule greatly expressed in RA-FLSs. The use of miR-483-3p mimics is associated with the greater production of IL-33 in RA-FLSs through the ERK pathway [102]. Subsequently, released IL-33 can interact with other immune cells to further regulate immune responses. The stimulation of peritoneal macrophages with IL-33 enhances the mRNA expression of MCP-1, IL-1β, and p-P65. Interestingly, a positive correlation between IL-33 and an anti-inflammatory IL-10 has been observed, which could suggest that, despite inducing inflammatory responses, IL-33 also enhances anti-inflammatory reactions [103]. Furthermore, macrophages respond to pro-inflammatory stimuli by secreting IL-33. The synovium of RA patients is associated with a greater presence of macrophage migration inhibitory factor (MIF) [104], a pro-inflammatory molecule that induces the expression of IL-33 in PBMC obtained from RA patients [105]. Moreover, IL-33 affects the behavior of dendritic cells, whose greater presence is found in the synovial fluids of RA patients [106]. This alarmin might modulate the functionality of dendritic cells to enhance the differentiation of Th17 cells [107], thus contributing to the progression of RA. IL-33 also enhances the migration of neutrophils through the CXCL1, CCL3, IL-1β, and TNFα mediators [108].

In addition, neutralizing IL-33 and the effect of systemic treatment further confirm its involvement in the pathogenesis of the disease. Despite ST2, there is a soluble form of the receptor (sST2) that can be used as a decoy for IL-33. In the CIA mouse model, the use of sST2 is associated with suppressed RA progression and reduced inflammation [109]. Moreover, treatment with TNFα inhibitors significantly reduces serum levels of IL-33 in responders [110,111], suggesting that IL-33 could be monitored to evaluate responses in patients treated with biologic agents, which has also been demonstrated in the case of rituximab [112]. A summary of the role of alarmins in the pathogenesis of RA is presented in Table 1.

## 5. Alarmins and Osteoarthritis

### 5.1. High-Mobility Group Box 1

Osteoarthritis is a degenerative chronic joint disease. Aging populations and a rising number of metabolic diseases are associated with an increasing number of OA patients. The clinical course usually involves the loss of joint function and pain. Multiple risk factors are associated with the development of OA, including age, obesity, prior joint trauma, genetic factors, and female sex [113]. It has been reported that OA affects nearly 27 million Americans, and this number is expected to double by 2030 [114,115]. This disease frequently involves the knee and hip joints, as well as the spine [116,117]. Articular cartilage is involved in smoothing the surface of the joint. Together with synovial fluid, these elements decrease friction during joint motion. As OA progresses, the cartilage becomes weaker, the space between joints narrows, and osteophytes may appear on bone surfaces [118,119]. Recent studies have found that the pathogenesis of OA is complex and involves immune mediators, such as alarmins.

To begin with, higher gene and protein expression of HMGB1 and RAGE have been detected in the synovial tissues of OA patients [120]. Moreover, the elevated expression of this alarmin has been observed in OA chondrocytes [121]. Li and colleagues found higher synovial fluid concentrations of HMGB1 in OA patients and that these levels positively correlated with OA severity [122].

**Figure 3:**
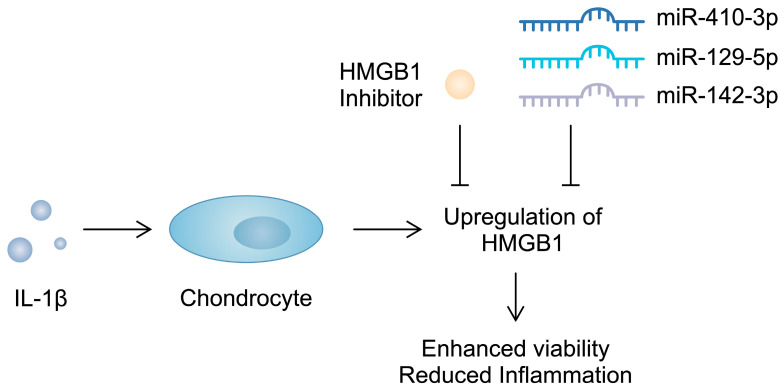
Molecules targeting HMGB1 were found to suppress pro-inflammatory responses in chondrocytes induced by IL-1β.

Importantly, apart from serving as a biomarker, HMGB1 contributes to the progression of OA. The intra-articular introduction of anti-HMGB1 antibodies significantly reduces the OARSI score in mice after anterior cruciate ligament transection (ACLT) [123]. Similarly, the use of glycyrrhizim, an HMGB1 inhibitor, is associated with the enhanced viability of IL-1β-pretreated chondrocytes. Importantly, this agent also suppresses the previously stimulated expression of inflammatory markers and cartilage-degrading enzymes [124]. Furthermore, several studies have demonstrated that non-coding RNA (ncRNA), molecules that regulate gene expression, can target alarmins and induce beneficial effects in chondrocytes. Specifically, miR-410-3p, miR-129-5p, and miR-142-3p have been found to directly target and downregulate HMGB1 in chondrocytes, which is associated with reduced inflammatory responses [125,126,127] (Figure 3). Consequently, the use of these molecules might be associated with protective mechanisms in OA. Importantly, in an in vivo experiment, the overexpression of miR-410-3p reduced cartilage damage in OA mouse models [125]. Additionally, an in vitro OA model is generated when chondrocytes are treated with IL-1β. During this process, an upregulation of HMGB1 can be observed, further confirming its involvement in the pathogenesis of OA [126]. Direct stimulation of mouse pre-chondral cell lines with recombinant HMGB1 has demonstrated a variety of effects induced by this alarmin. It stimulated the apoptosis of cartilage cells and enhanced the protein expression of a number of cartilage-degrading enzymes. Mechanistically, HMGB1 is associated with the Wnt/β-catenin signaling pathway; it enhances the expression of β-catenin [128]. Importantly, the enhanced activity of this pathway has been suggested to take part in the pathogenesis of OA, possibly through the upregulation of cartilage-degrading enzymes [129]. Therefore, HMGB1 seems to be involved in several mechanisms driving the progression of OA. Current evidence suggests that this alarmin enhances inflammatory responses and could be associated with the upregulation of cartilage-degrading enzymes, suggesting that HMGB1 could become a therapeutic target.

### 5.2. S100 Proteins

S100A8 and S100A9 are involved in the pathogenesis of OA by regulating the activation of synovium and the degradation of cartilage [130]. Both alarmins are overexpressed in synovial fluid in OA, and they are positively correlated with radiological joint damage [131,132]. Furthermore, serum levels of S100A8/A9 are positively correlated with clinical symptoms, such as knee pain, as well as the serum concentrations of enzymes associated with cartilage degradation [131,133]. S100A8/A9 and S100A6 are believed to be diagnostic markers of OA [134,135,136]. However, in a study by Mahler and colleagues, the authors did not prove that calprotectin is a biomarker in patients with hand, knee, or hip OA [137].

S100 proteins are associated with macrophage phenotypes and behavior, which may depend on the stage of differentiation. Specifically, S100A8 expression has been correlated with M2 macrophage infiltration [134]. The stimulation of synovial tissues with recombinant S100A9 enhances the mRNA expression of pro-inflammatory mediators such as IL-6, IL-8, and IL-1β [138]. However, during macrophage differentiation, exposure to S100A9 leads to enhanced differentiation toward M2-like macrophages. Interestingly, this alarmin enhances the expression of both pro-inflammatory and anti-inflammatory mediators, together with MMPs [139]. Zreiqat et al. demonstrated that S100A9 from articular chondrocytes is involved in the early processes of cartilage degradation through the promotion of aggrecanases and MMPs. However, the authors found that they are not involved in the subsequent progression of OA [132]. Schelbergen et al. reported that S100A8/A9 can increase the formation of osteophytes and thus may be used as biomarkers of osteophyte development [140]. Similarly to HMGB1, S100A8 and S100A9 interact with the Wnt signaling pathway. Specifically, the intra-articular administration of S100A8 promotes canonical Wnt signaling. The enhanced expression of dickkopf-1 (Dkk-1) is suppressed, which is associated with suppressed signaling, resulting in the alleviated expression of MIP-1a, IL-6, and MMPs [141]. Importantly, the administration of adipose-derived stem cells in experimental OA reduces the levels of S100A8/A9 [142].

As the catabolic effects of S100A8/A9 are TLR-4-dependent [143], their interaction could be targeted. Paquinimod is an immunomodulatory drug that prevents S100A9 from binding to TLR-4. It suppresses the expression of IL-6, IL-8, and MMP1/3 in the human OA synovium. Importantly, prophylactic treatment with paquinimod reduces cartilage degradation and the formation of osteophytes [144].

The presence of S100A8/A9 is associated with several molecules implicated in the pathogenesis of OA. For instance, in a study by Corr and colleagues, the authors observed that basic calcium phosphate (BCP) upregulated the expression of MMP1 and S100A8 in macrophages [145]. BCP crystals are long-known contributors to OA development, as their presence correlates with radiographic progression. Mechanistically, BCP crystals enhance the production of IL-6 by chondrocytes, as well as osteoclastogenesis [146]. Similarly, alarmins seem to be involved in the biological effects induced by IL-22, a mediator that has been suggested as a therapeutic target in OA [147]. Specifically, IL-22 has been found to enhance the production of MMP-1 and calprotectin [78]. Overall, these studies demonstrate the involvement of S100 proteins in mechanisms and molecules associated with OA progression.

### 5.3. Interleukin-33

Few studies have investigated the presence and role of IL-33 in the context of OA. To begin with, a higher expression of the IL-33 gene has been observed in articular chondrocytes from OA patients, as compared with healthy controls [148]. Interestingly, IL-33 may be associated with vitamin D. Specifically, greater immunoexpression of IL-33 has been observed in vitamin D-deficient swine. Interestingly, in vitro experiments using human articular chondrocytes have shown that calcitriol could reduce the expression of IL-33, along with other pro-inflammatory mediators, their receptors, and MMPs [149]. Similarly, by using human chondrocytes, He et al. observed that recombinant IL-33 increases the gene and protein expressions of cartilage-degrading enzymes (ADAMTS-5; MMPs) and reduces the expression of chondrogenic factors such as aggrecan, COL2A1, and SOX-9. Importantly, the authors demonstrated the importance of IL-33 in the pathogenesis of OA, as a cartilage-specific knockout of this alarmin was associated with reduced pain, cartilage degradation, and synovitis in mice. Similar findings have been observed after introducing the ST2-neutralizing antibody [150]. The pro-inflammatory effects of IL-33 affect anti-inflammatory mediators, as the stimulation of chondrocytes with IL-33 reduces the expression of IL-37, a cytokine that has been suggested to be used in the treatment of OA [151,152]. Interestingly, IL-33 may affect the biological effects of other alarmins since it upregulates the expression of the RAGE receptor in chondrocytes [151]. Therefore, current evidence suggests that targeting IL-33 may be associated with reduced inflammation and the suppressed progression of OA. A summary of the role of alarmins in the pathogenesis of OA is presented in Table 2.

## 6. Alarmins and Psoriasis

### 6.1. Typical Features and Epidemiology of Psoriasis

Psoriasis is a chronic inflammatory skin disease that lowers quality of life and is characterized by keratinocyte hyperproliferation and erythematous scaly plaques. It can develop in any part of the body. However, the most frequently affected are parts at risk of frequent injuries, such as elbows, knees, or loins. Psoriasis affects up to 3% of the world’s population, women and men equally, with variations in different regions. The disorder has a bimodal age distribution ranging from 18 to 39 years and 50 to 69 years [153].

Trauma to the skin is an important trigger that may precipitate the appearance of psoriatic lesions. It is widely known as Koebner’s phenomenon, and the triggering of the local inflammatory process is through the release of a nerve growth factor, a molecule that has been suggested to be involved in the pathogenesis of psoriasis. In addition, air pollution and bacterial, fungal, and viral infections are also linked to psoriasis. Despite the abovementioned factors, behavioral factors like dietary habits, smoking, and alcohol consumption are key risk factors. Regarding immunology, the skin is a fascinating milieu where the immune system is in a tight relationship with the endocrine and nervous systems. Consequently, mental status significantly influences the course of psoriasis, especially disorders like depression, anxiety, and chronic stress. Importantly, several drugs have been found to aggravate or induce the onset of psoriasis. These agents include beta-blockers, calcium-channel blockers, thiazide diuretics, lithium, anti-malarial drugs, interferons, imiquimod, angiotensin-converting enzyme inhibitors, terbinafine, tetracycline, NSAIDs, and fibrates [153]. Approximately 20% of patients with psoriasis develop psoriatic arthritis, further disfiguring and disabling them. These patients are also more often affected by other autoimmune diseases, such as rheumatoid arthritis, systemic lupus erythematosus, Sjogren syndrome, ulcerative colitis, vitiligo, and inflammatory bowel disease [154,155,156,157,158,159].

Studies claim that the pathogenesis of psoriasis is a combination of genetic predisposition with certain environmental factors. The evidence from the literature supports three main hypotheses on the origin of psoriasis; the first one considers it to be an autoimmune process; the second involves dysbiosis in the skin, as Toll-like receptors (TLR) have been found to be overexpressed in the keratinocytes and dendritic cells of psoriatic skin. TLRs induce the synthesis of antimicrobial peptides, leading to the activation of Th1 and Th17, which then release IL-18 and IL-23, respectively. The last theory assumes that an imbalance between oxidant and antioxidant mechanisms leads to the dysregulation of the immune system [153].

### 6.2. Pathogenesis of Psoriasis

Chronic inflammation involving an interplay between cytokines, chemokines, reactive oxygen species (ROS), antimicrobial peptides, and autoantigens is the main underlying mechanism in the pathogenesis of psoriasis. Inflammatory processes driving the progression of this disease involve both innate and adaptive immunity. Furthermore, a large number of cells are implicated in its pathogenesis. Keratinocytes are major responders to the skin’s inflammatory mediators, but other cells, such as dendritic cells, macrophages, mast cells, and neutrophils, are also involved in its pathogenesis. Their defensive role relies on phagocytosis and the presentation of antigens. These processes lead to the release of inflammatory mediators: IL-6, Il-1-β, TNF-α, IL-23, IL-12, CCL20, CXCL5, CXCL8, CXCL9, CXCL10, prostaglandins, leukotrienes, histamine, and ROS. Antimicrobial peptides, e.g., LL-37, beta-defensins, and S100 proteins, also play a key role in psoriasis, contributing to epidermal hyperproliferation, angiogenesis, and the activation of other cells of the immune system. Under pro-inflammatory conditions and the influence of IL-23 secreted by dendritic cells, naïve Th cells differentiate into Th17 and Th22 cells. Subsequently, these cells secrete IL-17A and IL-22, which enhance the migration of immune cells into the site of inflammation but also stimulate hyperproliferation and alter the maturation of keratinocytes. Moreover, IFN-gamma further stimulates antigen-presenting cells into producing pro-inflammatory mediators, which maintain the ongoing inflammatory process in the skin [153]. Regulatory T cells (Tregs) are also involved in the etiology of psoriasis. Their primary role is to inhibit the immune response and thus prevent the development of autoimmune diseases. However, the protective function of Tregs, both in the circulation and the skin, is impaired in patients with psoriasis, which inevitably alters the Th17/Treg balance [160].

### 6.3. High-Mobility Group Box 1

We previously mentioned that HMGB1 and its receptors are implicated in the pathogenesis of RA and OA. Importantly, current evidence suggests that HMGB1 also contributes to the progression of psoriasis. Higher levels of the alarmin have been noted in the serum of psoriasis patients [161,162]. Furthermore, a higher expression of this molecule in lesional skin, as compared with adjacent healthy tissue, has been detected. HMGB1 serum levels have been positively correlated with disease severity, as measured by the PASI score [162,163]. Moreover, the systemic treatment of psoriasis reduces the expression of HMGB1, which strongly suggests its involvement in the underlying mechanisms leading to the development of psoriasis [164]. Interestingly, different expression profiles of HMGB1 might point to different types of psoriasis. For instance, higher expression of this alarmin has been observed more among patients with generalized pustular psoriasis than those with psoriasis vulgaris. However, the difference was insignificant when serum levels of HMGB1 were compared [164]. Additionally, in an intriguing study by Mohamad et al., the authors found that patients with higher levels of HMGB1 had three vascular patterns of plaques when examined under dermoscopy and were more likely to have dotted and globular or looped vascular patterns, as well as ‘thick’ and ‘coarse’ scales. However, no significant difference was observed in scale distribution [165]. The described association between HMGB1 and vascular patterns provides an important correlation between HMGB1 levels and the severity of psoriasis, as the increase in the number of vascular patterns reflected the psoriasis severity.

Under normal circumstances, HMGB1 is expressed in the nucleus of epidermal cells, while in psoriasis vulgaris, it is located in the cell’s cytoplasm. According to previous data, HMGB1 translocates to the cytoplasm in response to different factors, such as LPS, TNF-alfa, and IFN-c [164]. This agrees with a previous study by Chen et al., who suggested that the relocalization of HMGB1 to the cytoplasm represents the signal for its secretion [162]. Secreted HMGB1 takes part in intercellular communication, which affects the functionality of immune cells. Interestingly, a significant relationship exists between HMGB1 and psoriasin (S100A7), which might suggest that they are released together [161]. Psoriasin is an antimicrobial peptide released by keratinocytes and leukocytes stimulated with IL-17, IL-22, and TNF-α. Its role involves chemotaxis for granulocytes, monocytes, and lymphocytes, and it is also said to contribute to psoriasis angiogenesis [166].

The secretion of HMGB1 affects keratinocytes by promoting their excessive proliferation and the expression of inflammatory cytokines, enhancing a vicious cycle. These observations confirm the involvement of both HMGB1 and keratinocytes in the pathogenesis of psoriasis, supporting the idea that keratinocytes secrete pro-inflammatory factors and respond to them [167]. Furthermore, as demonstrated in confocal fluorescence, HMGB1 co-localizes with keratins 1 and 10, molecules implicated in the pathogenesis of psoriasis, as these keratins are the markers of the early differentiation of keratinocytes [168]. Another mechanism in which HMGB1 is implicated is autophagy, a cellular recycling process. Wang et al. found a significant association between functionally active autophagy and psoriasis severity. Specifically, cytosolic HMGB1 acts as a Beclin 1-binding protein, thereby promoting autophagy. Beclin-1 (BECN1) plays an important role in autophagic programmed cell death, and its expression has been found to be higher in psoriatic epidermises than in healthy controls [169]. Therefore, the results of the study by Wang et al. strongly implicate the role of HMGB1 in autophagy as a crucial part of psoriasis etiology. Moreover, HMGB1 is more effective than other autosecretory proteins in regulating psoriasiform cutaneous inflammation. The specific ablation of HMGB1 in keratinocytes attenuates imiquimod-induced skin inflammation in a mouse model [169].

**Figure 4:**
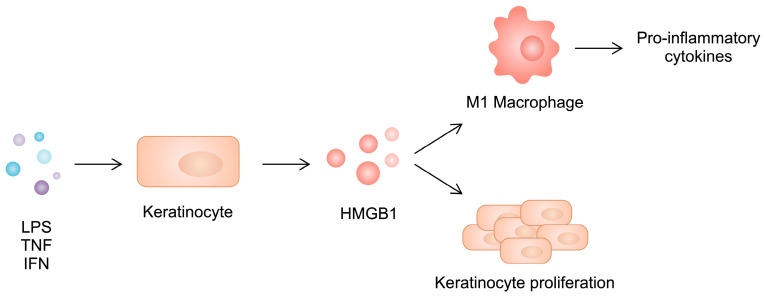
Stimulated keratinocytes secrete HMGB1, which induces keratinocyte proliferation and enhances macrophage polarization toward the pro-inflammatory phenotype.

Similarly to RA, there is an imbalance between macrophage phenotypes in psoriatic skin. Specifically, a greater presence of the pro-inflammatory M1 subtype can be observed. HMGB1 released from keratinocytes shifts the polarization of macrophages toward the inflammatory phenotype, further enhancing the production of inflammatory cytokines such as IL-17, IL-1β, TNF-α, IL-12, and IL-6. At the same time, silencing HMGB1 in psoriatic keratinocytes inhibits the development of the M1 phenotype, diminishing their cytokine production (Figure 4). Interestingly, the M2 type is not affected. However, the level of IL-10 increases in M2 macrophages, with unchanged expression of TGF-β.

As previously mentioned, systemic treatment is associated with a reduction in HMGB1 expression. In animal models, Li et al. aimed to confirm whether the therapeutic effect of etanercept affected the HMGB1 signaling pathway. The authors found that administering etanercept decreased the protein expression of HMGB1 but also that of RAGE, TLR4, and p-P65. Overexpression of HMGB1 attenuated the inhibitory effect of etanercept. Mechanistically, TNF-α causes HMGB1 translocation to the cytoplasm, which, as mentioned before, is a signal for its secretion, enabling this alarmin to exert immune effects [170]. One study examined the level of alarmins after anti-psoriatic therapy in pediatric patients who underwent the Goeckerman regimen (GR), which consists of applying pharmaceutical crude tar and UVB light exposure. Administering GR resulted in a significant drop in HMGB1, together with a reduction in the PASI score [171].

### 6.4. S100 Proteins

S100 proteins exert multiple intra- and extracellular functions, such as initiating and maintaining inflammation, cell differentiation, proliferation, apoptosis, and microbial resistance. In psoriatic skin, higher expression of S100A2, S100A7, S100A8, S100A9, S100A12, and S100A8/A9 has been observed [161,172,173,174,175,176,177]. Furthermore, in the sera of psoriatic patients, the levels of S100A7, S100A8/A9, and S100A12 are increased compared with control groups [161,174]. Moreover, S100A7 and S100A12 have been positively correlated with the PASI score in patients with a severe disease course [161,166,174].

Studies suggest that S100 proteins are involved in the processes associated with the impaired skin barrier, as well as inflammatory responses. Laboratory studies investigating its underlying mechanism discovered the involvement of S100 family genes [173]. Specifically, a reduction in hydration stimuli decreased the expression of genes associated with epidermal barrier function and increased that of S100 family genes. Moreover, administering exogenous S100A8 and S100A9 decreased the expression of filaggrin and loricrin, proteins crucial for sustaining the skin barrier [173], indicating important interactions between S100 family members and proteins associated with the skin barrier.

Regarding inflammatory responses, S100 proteins are involved in feedback loops with cytokines involved in the pathogenesis of psoriasis. Firstly, the induced expression of S100A8/S100A9 results in an increase in the transcriptional level of IL-17A, mediating the development of autoreactive CD8+ T cells. In turn, IL-17A, together with IL-17F, IL-1α, and TNF, upregulates genes encoding both S100A8 and S100A9, and this induction is the most pronounced in the early stages of keratinocyte maturation [172]. In another study, S100A9 was positively correlated with the expression of Th17-related mediators, including IL-1-β, IL-6, IL-21, IL-22, IL-27, TNF, IL-12-β, IL-23A, and IL-17A [178]. In turn, IL-22 induces the expression of acute inflammatory proteins, which are associated with the upregulation of S100A9 in psoriatic skin [179]. Furthermore, S100A8 and S100A9 have been found to be associated with the expression of IL-8 and MCP-1 [180]. These pro-inflammatory responses could be associated with increased NF-κB activity [181]. Interestingly, in a study by Christmann et al., the authors showed that S100A8 and S100A9 do not induce keratinocyte differentiation or inflammatory responses. Rather, these proteins act as additional inflammatory mediators during the inflammatory process in the skin, mediated by IL-17, amplifying disease activity in psoriasis suggesting that these proteins may be involved in more complex signaling pathways. Nevertheless, the knockout of S100A9 is associated with the attenuation of psoriasis-like disease in mice, which further confirms its involvement in the pathophysiological mechanisms associated with this skin condition [172].

Apart from keratinocytes, monocytes represent another cell group involved in feedback loops between cytokines and alarmins. IL-6 is upregulated in psoriasis and is crucial to inflammatory crosstalk and pathways, acting synergistically with IL-1 and TNF-α. The effect of IL-6 on the induction of gene expression in monocytes is higher after stimulation with proteins from the S100 family [172]. A study by Berg et al. showed that S100A8 and S100A9 act not only locally but also play an important role in high-risk coronary plaque disease in patients with psoriasis and show a positive linear relationship with lipid-rich necrotic core (LRNC). Calprotectin causes surrounding neutrophils and monocytes to secrete NF-κB pathway products and drive the adhesion and migration of granulocytes through the endothelium. Fortunately, a therapy with biologics is associated with a 79% reduction in S100A7, S100A12, and S100A8/A9, as well as LRNC [176].

The treatment of psoriasis affects the expression of S100 proteins. Firstly, Benezeder et al. carried out a clinical trial aiming to elucidate the mechanism of action of topical dithranol (anthralin). The authors revealed that this caused a rapid decrease in S100A7 and S100A12 [182]. In another study, administering the previously mentioned GR regimen resulted in a significant drop in S100A7 and S100A9 [171]. Kruger et al. observed that treatment with the anti-IL-17a agent secukinumab decreased the expression of S100 proteins, further confirming the influence of IL-17 on the S100 protein family [172,183]. Similar effects have been observed after treatment with risankizumab [184], tacrolimus [185], and etanercept [174]. Additionally, the use of the novel TYK2 inhibitor deucravacitinib is associated with reducing the expression of S100A9 and S100A8 to that of healthy skin [186].

### 6.5. Cathelicidin

Cathelicidins are a family of small antimicrobial peptides (AMPs). They are an inseparable component of skin immunity, exhibiting a broad spectrum of antimicrobial activity against bacteria, viruses, and fungi. However, their role is not solely limited to defense against pathogens; they can trigger specific defense responses in the host [153]. Cathelicidin, also known as LL-37, comes from its precursor—hCAP18 (human cationic antimicrobial protein 18)—encoded by a single cathelicidin antimicrobial peptide gene (CAMP) in humans. Proteases cleave the hCAP18 into several active AMPs, including a 37-residue-long peptide cathelicidin, hence its abbreviation—LL-37 [153]. In 2014, cathelicidin was the first autoantigen discovered in psoriasis and remains the most widely researched one. At the same time, it was the first AMP in mammalian skin to be described [187].

Studies have proven the increased expression of LL-37 in the epidermis and dermis of psoriatic skin [188]. The expression of IgG autoantibodies against LL-37 autoantigen has been observed in the sera of patients with psoriasis, and this correlates with the PASI score. Also, it reflects disease progression in longitudinally collected serum samples from patients with psoriasis [189]. Autoantibodies against LL-37 are even higher in the sera of patients with psoriasis with psoriatic arthritis than in patients with only skin manifestations [189,190]. The role of LL-37 is not only limited to the permanent stimulation of innate immune cells but is also recognized as an autoantigen, circulating T cells in up to 75% of patients with moderate-to-severe psoriasis [188,190,191]. Whether cathelicidin comes from infiltrating neutrophils is still a subject of discussion; it is also thought to be produced by keratinocytes [192].

The role of LL-37 in the pathogenesis of psoriasis is exerted by activating dermal dendritic cells (dDCs) via TLR7 and TLR9. Upon the injury of keratinocytes, LL-37, self-DNA, and RNA are released [193]. Subsequently, cathelicidin can bind the nucleic acids and form a complex that activates LL-37-specific dDCs [188,194]. That action enables the whole inflammatory cascade, as activated dDCs produce excessive amounts of IFNα that can further activate conventional dendritic cells and other immune cells in the dermis [194,195]. At the same time, dermal dendritic cells release pro-inflammatory cytokines in lymph nodes such as IFNα, IL-6, IL-1β, TNFα, IL-12, and IL-23 [196,197]. Moreover, LL-37 has been shown to induce the proliferation of circulating CD3+ T cells in 24 out of 52 psoriasis patients. The vast majority of LL-37-reactive CD3+ T cells produce IL-17, and the capacity of their IL-17 production is associated with disease severity [198,199].

### 6.6. Heat Shock Proteins

The small heat shock protein (HSP) family includes molecules involved in cell survival, cytoskeleton dynamics, and cell differentiation. HSPs exhibit housekeeping and homeostatic functions in cellular stress. They are both pro-apoptotic and anti-apoptotic, depending on the conditions, and also play a crucial role in resistance to heat shock and oxidative treatments [200].

The psoriatic epidermises contain abundant levels of HSP27, HSP60, HSP70, and HSP90α [200,201]. Additionally, a higher expression of HSP27 has been observed in psoriatic patients with comorbid metabolic syndrome [202]. Serum analysis of psoriatic patients has revealed significantly greater concentrations of anti-Hsp90α antibodies compared with healthy controls. Moreover, the level of anti-Hsp90α antibodies positively correlates with the PASI score [203]. It has been found that 47% of psoriasis patients demonstrate significantly elevated antibody titers to HSP65. Additionally, a previous study demonstrated a positive correlation between antibody activity toward HSP65 and psoriasis disease activity [200].

Hsp27 is uniformly distributed throughout the healthy epidermis. In psoriatic skin, however, it is abundantly elevated. Owing to the molecular mimicry between human and microbial HSPs, the immune reaction against a microbial one may involve an attack against autologous HSPs, leading to autoimmunity [153]. HSP27 may also be an autoantigen associated with immune responses in streptococcal-induced psoriasis [204]. Moreover, studies have observed increased HSP27 expression in response to stress, explaining the role of a stressful environment in exacerbating psoriatic lesions and discovering antigenic determinants underlying its mechanism [153]. However, the data are inconsistent, as the phosphorylation of HSP27 by p38-MAPK is required for keratinocyte differentiation, and HSP27 inhibits the expression of IL-8 and PGE2 stimulated by TNF-α through NF-κB signaling. In addition, UV treatments upregulate HSP27 mRNA, suggesting the protective role of HSP27 in keratinocytes.

HSP90 is the most abundant chaperone protein in eukaryotic cells, playing an essential role in stress tolerance. It controls cellular processes such as hormonal signaling, cell cycle control, and the activation of many regulatory proteins [203]. HSP90 is prevalent in normal-appearing skin; however, certain isoforms, such as HSP90α, are overexpressed under stressful conditions [205]. As previously mentioned, Th1 and Th17 cytokines are involved in the pathogenesis of psoriasis. Act 1 adaptor protein (Act 1) is a molecule that is essential in IL-17-dependent signaling. HSP90 plays a key role in regulating Act 1, resulting in the facilitation of IL-17A signaling [206].

HSP70 is a family of proteins that are constitutively expressed in the skin. Previous studies have revealed that the HSP70 family can rescue DNA breaks from ROS-induced insults [200]. CD91 is one of the HSP70 receptors and is expressed predominantly by activated antigen-presenting cells (APCs). The accumulation of cells expressing CD91 has been observed in the early stages of psoriatic lesions [207]. However, some studies have revealed lower levels of HSP70 in basal, suprabasal, and superficial epidermal layers of psoriatic lesions when compared with those of normal skin [208]. The role of HSP70 in developing psoriasis seems to confirm the ongoing discussion on the association of the disease with *Malassezia furfur*. Baroni et al. demonstrated an increased level of HSP70 in *Malassezia furfur*-positive patients with psoriasis [209].

The role of HSP72 in psoriasis needs more elucidation as, in one study where systemic inflammation was induced by LPS, HSP72 induced by thermal pre-treatment inhibited IL-6 and TNF-α. When compared with healthy skin, both normal and psoriatic skin, when heat-stressed, showed higher levels of HSP72 in basal- and suprabasal-layer cells [200]. Moreover, the immunological role of HSP65 is of great importance [200]. Interestingly, HSP65 is also an antigen of *Mycobacteria* species, supporting the theory of molecular mimicry and underlying infection-induced psoriasis. Another example of linking immunological reaction to microbial factors and the pathogenesis of psoriasis was presented by Cancino-Diaz et al. The authors suggested the role of *S. pyogenes* in the pathogenesis of psoriasis as they observed an association between a high response to *S. pyogenes* HSP60 and the chronic form of psoriasis [210].

As members of the HSP family may be involved in the pathogenesis of psoriasis, several studies have investigated whether they could become therapeutic targets. Seifarth et al. found that the treatment of animal models with alfalfa-derived HSP70 had an anti-psoriatic effect, resulting in significantly lower PASI scores in treated individuals. Moreover, the therapeutic effect was exerted at the gene level, as alfalfa-derived HSP70 downregulated IL-4, IL-5, and IL-17F but it upregulated IL-17A and IL-22 [211]. A study by Tukaj et al. demonstrated that immunization with HSP70 led to clinical and histological improvement in imiquimod-induced, psoriasis-like skin inflammation in mouse models. At the same time, administering anti-HSP70 antibodies resulted in lower disease activity and the reduced presence of Th17 cells [212]. Furthermore, the use of RGRN-305, an HSP90 inhibitor, was associated with the downregulation of the TNF, IL-1β, IL-6, and CXCL8 in keratinocytes and mouse models. Mechanistically, the anti-inflammatory effect was suggested to occur due to the suppression of the NF-κB, ERK1/2, p38, and c-Jun signaling pathways [213]. The expression of HSP90 has also been analyzed after administering the widely used anti-psoriatic drug ustekinumab. A significant downregulation of HSP90 was observed after introducing treatment in [214]. In another study, in which an oral HSP90 inhibitor Debio 0932 was studied, it reduced epidermal thickness in a psoriasis xenograft transplantation model [215].

### 6.7. Defensins

Defensins are cationic microbial peptides. Like cathelicidin, they are expressed as propeptides [55]. However, in contrast to cathelicidin, which is encoded by a single gene, defensins have multiple genes that form several gene clusters. Defensins are host defense peptides involved in antimicrobial and immune signaling activities. Six human alpha-defensins (human neutrophil peptides 1-6 (HNPs)) and four human beta-defensins (hBDs 1-4) have been identified. They are produced by neutrophils and Paneth cells. Furthermore, mechanical stress increases the production of hBDs [216]. Other studies have shown that specific β-defensins might be endogenous pruritogens, directly stimulating sensory neurons [217].

In psoriatic skin, only HNP1-3, hBD-2, and hBD-3 are recognized [218,219,220]. In a study by Jansen et al., the authors found that hBD-2 serum levels were 400-fold higher in severe psoriasis than in healthy controls [221,222]. Furthermore, the skin of psoriatic patients showed higher hBD-2 expression than that of healthy subjects [223]. Their high expression may account for a lower prevalence of skin infections in psoriatic patients. Importantly, the risk for psoriasis is significantly associated with higher genomic copy numbers of hBD genes [224]. The expression of beta-defensins in psoriasis lesional skin is induced by TNF-α and IFN-gamma. Moreover, TNF-α promotes the secretion of hBD-2 synergistically with IL-17 via the induction of transcription factors (OCT-1, Nf-κB, and AP-1) [225]. Hence, it has been suggested that hBD is a marker of IL-17A-related skin diseases, thus predicting the response to anti-IL-17A treatment [226]. The role of hBD-2 in psoriasis, although not fully understood, can be attributed to the ability to act as a ligand for chemokine receptor 6 (CCR6), thereby inducing Th17.

In a randomized, placebo-controlled trial, a profile of gene expression after treatment with deucravacitinib in patients with moderate-to-severe plaque psoriasis was analyzed. The study revealed a return to non-lesional levels of beta-defensin 4A/B [186], and the impact of treatment was dose-dependent. Also, studies of patients receiving secukinumab or guselkumab have revealed a reduction in the expression of hBD2 in the skin [183,227]. However, not all studies are coherent regarding secukinumab’s effect on the level of this alarmin. A study by Wu et al. showed higher serum levels of hBD2 after treatment with secukinumab than in a normal population [228]. Another study by Cardner et al. analyzed the serum levels of beta-defensin in 2000 patients with psoriatic arthritis in a placebo-controlled phase-III clinical trial of secukinumab. It showed the baseline level of beta-defensin was quantitatively associated with the clinical response to secukinumab, as well as to adalimumab [229]. Also, treatment with risankizumab resulted in a decrease in hBD2 [184]. By contrast, treatment with narrow-band ultraviolet B (nb-UVB), a prevalent form of psoriasis treatment, did not affect the levels of hBD1 and hBD2, suggesting its mechanism of action is independent of influence on defensins [230].

### 6.8. Thymic Stromal Lymphopoietin

Thymic stromal lymphopoietin (TSLP) is an epithelial-derived cytokine from the IL-2 family resembling IL-7. It is known for promoting type-2 immune responses and being involved in many Th2-oriented diseases. The TSLP receptor (TSLPR) is expressed in the skin, gut, and lungs. After binding to the receptor, JAK1 and JAK2 are recruited and phosphorylated, and downstream STAT5 is activated. Consequently, the expression of pro-inflammatory genes is induced [231]. TSLP has two isoforms—the anti-inflammatory short-form TSLP (sfTSLP) and the pro-inflammatory long-form TSLP (lfTSLP). While sfTLSP is expressed constitutively, the lfTSLP is upregulated only in an inflammatory environment [231].

Several studies have examined the role of TSLP in the context of psoriasis. Firstly, serum levels of TSLP are significantly elevated in patients with psoriasis, and its concentrations are positively correlated with the PASI score. Furthermore, a greater expression of TSLP can be detected in psoriatic keratinocytes [227,232]. However, Suwarsa et al. found no significant difference between TSLP expression in the lesional vs. non-lesional skin of psoriasis vulgaris patients [233]. Also, the level of TSLP was higher in patients with psoriasis with psoriatic arthritis and smokers [232]. In addition, synovial fibroblasts from patients with PsA have shown elevated TSLP expression [234].

TSLP is produced by mutant bulge hair follicle stem cells and DCs, both dermal and myeloid, in patients with psoriasis [235]. Additionally, TSLP is expressed by keratinocytes [236]. This alarmin can stimulate the hyperproliferation of adjacent epidermal cells and induce the expression of VEGF-α, contributing to inflammation and epidermal hyperplasia in psoriatic skin, demonstrating that it can act in an autocrine manner [236]. TSLP is also responsible for the maturation of antigen-presenting cells and plays an essential role in promoting IL-23 production via CD40L.

Therefore, it is speculated that, by blocking TSLP, the DC activation and production of IL-23 can be hindered [231]. The injection of antibodies against TSLP in psoriasis-like mouse models has resulted in the regression of psoriasis, reduced epidermal hyperplasia, decreased VEGF α expression, and the epidermal inhibition of STAT-5 phosphorylation [236]. One of the possible directions of therapeutic strategies is the suppression of TSLP gene activation by ghrelin, which acts through the activation of glucocorticoid receptors [237]. Tashiro et al., studying the effects of hypoxia on TSLP, found an inhibition of the TNF-α -induced expression of TSLP in human and mouse keratinocyte cell lines. However, the expression of TNF-α, IL-6, IL-8, MCP-1, and VEGF-A was not inhibited. Interestingly, the inhibition of TSLP was reversed by introducing an HIF-2α antagonist but not by an HIF-1α inhibitor. Considering the abovementioned findings, HIF-2α may be a new future therapeutic strategy for treating psoriasis [238]. As the use of TNF-α inhibitors is associated with an elevated risk of infections, targeting TSLP may be more suitable for treating psoriasis [239].

### 6.9. Interleukin-33

Studies have proven that patients with moderate-to-severe psoriasis have increased levels of IL-33 in their sera and greater expression intra-epidermally [240,241]. However, the serum level of IL-33 does not correlate with psoriasis severity [161]. Conflicting results have been published regarding the role of IL-33 in psoriasis. Firstly, in one study, injecting IL-33 into an imiquimod-induced psoriasis model improved the disease. The authors suggested that IL-33 plays an anti-inflammatory and protective role [241]. By contrast, another study found that a mouse model of imiquimod-induced psoriasis that lacked epidermis-specific IL-33 displayed much milder psoriatic lesions than control mice [242].

The role of IL-33 in the pathogenesis of psoriasis is exerted via the stimulation of keratinocytes to produce CXCL1 and CXCL8, responsible for recruiting neutrophils and CCL20, which recruits IL-17-producing T cells to the epidermis [243]. Moreover, IL-33 lets DC mature, which enables the induction of differentiation into Th17 cells, producing IL-17A, strongly involved in the pathogenesis of psoriasis, by stimulating keratinocytes into proliferation and causing epidermal thickening. Under the influence of IL-17A, keratinocytes produce CXCL8 and IL-36G, which induce neutrophil chemotaxis [244]. There are contrasting results on whether TNF-alpha induces the production of IL-33 in human keratinocytes [21]. However, it has been demonstrated that its production can be induced by INF-gamma. That process is mediated via the epidermal growth factor receptor (EGFR), ERK, and p38 pathways [245]. It has also been speculated that the production of IL-33 is dependent on JAK/STAT, as JAK inhibitors diminish IL-33 expression induced by IFN-gamma [20]. Moreover, it has been reported that IL-17 is involved in the induction of IL-33 expression via the EGFR, ERK, p38, and JAK/STAT1 pathways [240]. The literature also provides evidence of IL-4 and IL-13 involvement in stimulating IL-33 expression [246,247]. However, the JAK inhibitor does not affect the IL-4-induced production of IL-33, but ERK inhibitors have confirmed the expected results [247].

It is being debated whether mast cells contribute to the inflammatory process in psoriasis. Several studies concluded that the secretion of IL-1, IL-6, and IL-13 by mast cells is activated by IL-33, highlighting its role in psoriasis [248]. Furthermore, psoriasis is associated with pruritus, lowering the quality of life. However, it is not fully understood which cytokines are responsible for stimulating nerves, as the subcutaneous injection of IL-17A, IL-23, and TNF-α does not induce scratching behavior [20]. However, IL-33 has been found to directly stimulate nerves in psoriasis, confirming that it is involved in the induction of pruritis [20]. Moreover, according to a study on atopic dermatitis, scratching damaged keratinocytes further secreted IL-33 in cutaneous lesions [243]. IL-33 could trigger Th2 cells to produce IL-4, IL-13, and IL-31 [249], which, along with IL-33, decrease filaggrin and loricrin expression in keratinocytes, resulting in an impaired skin barrier [20]. In psoriatic arthritis, IL-33 has been suggested to be involved in the stimulation of osteoclastogenesis, a process of monocyte differentiation toward osteoclasts [250]. The treatment of psoriasis affects the expression of IL-33. Specifically, Meephansan et al. observed that MTX treatment was associated with the reduced expression of IL-33. By contrast, NB-UVB therapy led to an increase in thin alarmin [251].

### 6.10. Interleukin-1α

IL-1α is constitutively expressed in the epidermis as a proIL-1α, playing a crucial role in skin homeostasis. It can be found in keratinocytes, Langerhans cells, and dermal fibroblasts. However, because the skin is made mostly of keratinocytes, these cells are the main source of that cytokine, making the skin the prime tissue for producing IL-1. Inflammatory stimuli further enhance the expression of IL-1α [18].

The mature form of IL-1 is secreted with the help of inflammasome and caspase-1. When hypoxia, injury, or acidosis leads to cell necrosis, its whole content with both proIL-1 and IL-1α is released. When localized extracellularly, proIL-1α recruits neutrophils and monocytes as an initial step of inflammation. Other factors known to dysregulate IL-1 production in the skin are UV light, HPV infection, and disruptions in the skin barrier [18].

The role of the IL-1 family in the pathogenesis of psoriasis has been widely discussed in the literature. It has been shown that IL-1 is a potent cytokine released by activated keratinocytes and that it can induce inflammation by regulating several genes in the skin [252,253]. IL-1 cytokines can also further stimulate the production of IL-6, TNF, and IL-33 by mast cells [254]. Furthermore, IL-1α acts synergistically with IL-17A, IL-22, OSM, and TNFα in inhibiting the differentiation of keratinocytes, being a fundamental part of the disease’s etiology [255]. Moreover, IL-1 stimulates macrophages into releasing another member of the IL-1 family—IL-36, mediating innate and adaptive immune responses that lead to a chronic pro-inflammatory state. Additionally, IL-1 activates dendritic cells, which, in response, release IL-12 and IL-23, leading, subsequently, to the differentiation of Th1 and Th17 cells, playing an essential role in psoriasis pathogenesis [253].

Conflicting results have been published regarding the expression of IL-1α in patients with psoriasis. Romero et al. found a higher expression of IL-1α in psoriatic skin [256]. Studies have shown that keratinocytes express a receptor for IL-1 on their surfaces, which explains why these cells can respond to this extracellular cytokine. The abovementioned findings led Portugal-Cohen et al. to identify IL-1 as a disease biomarker [257]. Conversely, several studies have found that the expression of IL-1α is lower in lesional psoriatic skin than in non-lesional regions, both in adult and pediatric populations [258,259,260]. In addition, a moderate negative correlation between IL-1α and the PASI score has been observed [259]. Moreover, the level of IL-1α in the sera of psoriatic patients has been reported to be lower [261]. However, according to Mee et al., there is no significant difference in IL-1α expression between lesional and non-lesional biopsies from psoriasis patients, with a small decrease in only some of them [262].

Although more studies are needed to find out why the level of IL-1α is lower in lesional psoriatic skin, a possible explanation for this is the observed simultaneous increase in the IL-1RA-antagonizing inflammatory action of IL-1α. IL-1RA is produced by many cells that the skin is made out of, such as keratinocytes and dermal fibroblasts, as well as by neutrophils and macrophages infiltrating the skin. It is known that IL-1α, along with TNF-α, can stimulate the expression of IL-1R. Given the fact that glucocorticosteroids are effective in treating psoriasis and that they reduce the expression of TNF-α and IL-1, it is possible that glucocorticoids might affect the IL-1R-to-IL-1 ratio through the regulation of these cytokines [263,264,265,266]. Therefore, addressing the restoration of that ratio might be a promising target in strategies for future anti-psoriatic therapies.

New data have also emerged evaluating the level of gut cytokines in patients with psoriasis. IL-1 alpha has been found to be significantly elevated compared with healthy controls. However, a study by Yegorov et al. found no correlation between the gut IL-1 alpha level and the PASI score. This study’s results support the important role of gut dysbiosis and the gut–brain axis in modulating the inflammatory response of the skin’s immune system. Moreover, it may help to find a possible molecular mechanism underlying inflammatory bowel disease, a frequent comorbidity of psoriasis. However, more evidence is required to establish whether the intestinal mucosa is the origin of this inflammation or the consequence of the systemic inflammation present in psoriasis itself [267].

As a major inflammatory player, the level of IL-1α in anti-psoriatic treatments has been the subject of research. Treatment with anti-TNF-α, anti-IL-17A, and anti-IL-12/23 agents contributes to an increase in the average level of lesional IL-1α in the skin [259]. Mechanistically, it has been suggested that preformed IL-1α is stored in the deep layers of the epidermis and is used for restoring impaired barrier function. Moreover, as previously mentioned, IL-1 is constitutively expressed in the skin, especially during the regeneration process, implicating the important role of this alarmin in immune defense mechanisms [268]. In one study, administering MTX in an in vitro psoriasis model decreased IL-1α release by inhibiting transport mediated by solute carrier family 46 (SLC46). The results not only confirmed the effectiveness of treating psoriasis with MTX but also provided the underlying molecular mechanism involving IL-1α [269]. A summary of the role of alarmins in the pathogenesis of psoriasis is presented in Table 3, while the impact of agents used in the treatment of psoriasis on the expression and concentrations of alarmins is depicted in Table 4.

## 7. Conclusions

Alarmins are a broad group of molecules that stimulate inflammatory responses. They can serve as diagnostic biomarkers or could be used to monitor disease activity and treatment response. Furthermore, current evidence suggests that alarmins are involved in the pathogenesis of immune-mediated diseases, such as RA, OA, and psoriasis, and thus are potential therapeutic targets. However, the role of some of these proteins remains inconclusive. For instance, IL-33 has been found to induce both pro- and anti-inflammatory responses in the context of OA. Further studies are required to investigate pathways induced by alarmins and their involvement in inflammatory disorders.

## Figures and Tables

**Table 1:** Summary of mechanisms induced by alarmins that take part in the pathogenesis of rheumatoid arthritis.

Alarmin	Blood/Synovial Fluid Level/PBMC Expression	Synovial Expression	Potential Role in the Pathogenesis	References
HMGB1	Increased	Elevated in untreated patients compared with patients receiving methotrexate	HMGB1 may promote RA-FLS proliferation, migration, invasiveness, and the expression of MMPs. HMGB1 synergizes with LPS to induce aggressive behavior in RA-FLSs. HMGB1 stimulates synovial angiogenesis. HMGB1 promotes the production of pro-inflammatory cytokines from macrophages. By forming a complex with CXCL12, HMGB1 enhances monocyte migration. HMGB1 promotes macrophage pyroptosis, a process that has been implicated in the pathogenesis of RA. HMGB1 enhances the Th17 cell population, which plays a role in RA pathogenesis.	[27,28,29,31,32,33,37,38,41,43]
S100A8/S100A9	Blood: Increased	-	Calprotectin is released in NETs and contributes to neutrophil activation. S100A8/A9 stimulates the production of pro-inflammatory mediators in monocytes. The expression of S100A8/A9 in RA-FLSs increases after stimulation with IL-22, a cytokine involved in the pathogenesis of RA.	[57,58,59,72,75,78]
S100A11	Synovial fluid: Increased (vs. OA)	-	S100A11 is released from neutrophils during NETosis, and this alarmin can contribute to the pro-inflammatory responses of other neutrophils.	[86,87]
S100A12	Blood: Increased Synovial fluid: Increased (vs. OA)	-	S100A12 enhances the infiltration of neutrophiles. S100A12 has been found to enhance osteoclastogenesis.	[88,89,91]
IL-33	Blood: Increased Synovial fluid: Increased (vs. OA)		IL-33 regulates inflammatory responses in RA-FLSs.	[94,95,100,101]

CXCL—chemokine (C-X-C motif) ligand; HMGB1—high-mobility group box 1; IL—interleukin; NET—neutrophil extracellular trap; RA-FLS—rheumatoid arthritis fibroblast-like synoviocytes.

**Table 2:** Summary of mechanisms induced by alarmins that take part in the pathogenesis of osteoarthritis.

Alarmin	Blood/Synovial Fluid Level/PBMC Expression	Expression in Synovial Tissues/Chondrocytes	Potential Role in the Pathogenesis	References
HMGB1	Increased	Increased	HMGB1 levels positively correlate with disease severity. Targeting HMGB1 was associated with beneficial effects in in vitro and in vivo studies. miR-410-3p targets and decreases the expression of HMGB1. Overexpression of miR-410-3p in OA mouse models is associated with reduced cartilage damage. Treatment of chondrocytes with IL-1β increases the expression of HMGB1. HMGB1 enhances the expression of β-catenin in the pre-chondral cell line. Wnt/β-catenin pathway is associated with the progression of OA.	[120,121,123,124,125,126,128,129]
S100A8/A9	-	Increased	Serum levels of S100A8/A9 are positively correlated with the WOMAC score. Treatment of synovial tissue with S100A9 enhances the expression of pro-inflammatory cytokines. S100A9 enhances the production of cartilage-degrading enzymes. S100A8 and S100A9 are involved in the processes of osteophyte formation. S100A8/A9 can enhance signaling through the Wnt pathway. Using an S100A9/TLR-4 inhibitor is associated with suppressed OA.	[131,132,133,138,139,140,141,144]
IL-33	-	Increased	Stimulation of articular chondrocytes with IL-33 is associated with elevated expression of pro-inflammatory mediators and cartilage-degrading enzymes. Chondrocyte-specific knockout of IL-33 and the use of ST2-neutralizing antibodies alleviates OA in mice. Chondrocytes treated with IL-33 show reduced expression of anti-inflammatory IL-37.	[148,149,150,151]

HMGB1—high-mobility group box 1; IL—interleukin; OA—osteoarthritis; ST2—suppression of tumorigenicity 2; WOMAC—Western Ontario and McMaster Universities Osteoarthritis Index.

**Table 3:** Summary of mechanisms induced by alarmins that take part in the pathogenesis of psoriasis.

Alarmin	Serum Expression	Keratinocyte Expression	Potential Role in the Pathogenesis of Psoriasis	References
HMGB1	Increased	Increased	HMGB1 promotes the excessive proliferation and expression of inflammatory cytokines by keratinocytes. HMGB1 promotes autophagy. HMGB1 released from keratinocytes shifts the polarization of macrophages toward the inflammatory M1 phenotype.	[161,162,167,169]
S100A2	-	Increased	-	[177]
S100A7	Increased	Increased	-	[161,174]
S100A8	-	Increased	S100A8 decreases the expression of filaggrin and loricrin, impairing the skin barrier. S100A8 upregulates the expression of IL-6, IL-8, and MCP-1. Keratinocytes overexpressing S100A8 exhibit increased NF-κB activity.	[173,180,181]
S100A9	-	Increased	S100A9 decreases the expression of filaggrin and loricrin, impairing the skin barrier. S100A9 upregulates the expression of IL-6, IL-8, and MCP-1. IL-22 is associated with the upregulation of S100A9 in psoriatic skin. Keratinocytes overexpressing S100A9 exhibit increased NF-κB activity. S100A9 has a positive correlation with Th17-related gene expression, including IL-1β, IL-6, IL-21, IL-22, IL-27, TNF, IL-12-beta, IL-23A, and IL-17A.	[173,179,180,181]
S100A12	Increased	Increased		[161,174]
S100A8/A9	Increased	Increased	S100A8 and S100A9 increase the transcriptional level of IL-17A, mediating the development of autoreactive CD8+ T cells. S100A8 and A9 enhance the secretion of NF-κB pathway products from neutrophils and monocytes, contributing to plaque disease. In patients with psoriasis, S100A8/A9 potentiates the production of IL-8 induced by TNF-α.	[161,172,173,174,175,176,181]
Cathelicidin (LL-37)	Increased level of LL-37 autoantibodies	Increased	Cathelicidin is recognized as an autoantigen by circulating T cells. LL-37 activates dDCs via TLR7 and TLR9. Cathelicidin binds nucleic acids released from keratinocytes upon injury and forms a complex that activates LL-37-specific dDCs. LL-37 induces the proliferation of circulating CD3+ T cells, which produce IL-17.	[188,189,190,191,193,194,198,199]
HSP27	-	Increased	HSP27 may be an autoantigen associated with immune response in streptococcal-induced psoriasis. An increase in HSP27 expression in response to stress may explain the role of a stressful environment in exacerbating psoriatic lesions.	[153,204]
HSP60	-	Increased	High response to *S. pyogenes* HSP60 may suggest the involvement of that alarmin in the chronic form of psoriasis. An association between the response to *S. pyogenes* HSP60 and the chronic form of psoriasis has been observed.	[210]
HSP70	-	Conflicting results	HSP70 acts via its receptor—CD91—expressed predominantly by activated antigen-presenting cells, observed in the process of the early development of psoriatic lesions. HSP70 downregulates mRNA of IL-4, IL-5, and IL-17F and upregulates that of IL-17A and IL-22.	[207,208,211]
HSP90 α	Higher levels of anti-Hsp90α antibodies	Increased	The immune reaction against microbial HSP may attack autologous HSP, leading to autoimmunity. HSP90 plays a key role in regulating Act 1, a molecule that is essential in IL-17-dependent signaling.	[200,201,202,203,206]
HSP65	Elevated HSP65 antibody titers	-	HSP65 is an antigen of Mycobacteria species; it supports the mechanism of molecular mimicry and underlying infection-induced psoriasis.	[200]
Beta-defensins	Increased	Increased	Human beta-defensin 2 acts as a ligand for CCR6, thereby inducing Th17.	[221,222,223,226]
TSLP	Increased	Increased	TSLP stimulates the hyperproliferation of epidermal cells and their expression of VEGF-α. TSLP is responsible for the maturation of antigen-presenting cells. TSLP promotes IL-23 production via CD40L.	[216,227,231,232,233,236]
IL-33	Increased	Increased	IL-33 stimulates keratinocytes into producing CXCL1 and CXCL8, which are responsible for recruiting neutrophils, and CCL20, which recruits IL-17-producing T cells to the epidermis. IL-33 lets DC mature, which enables the induction of differentiation into Th17 cells, producing IL-17A. IL-33 activates the release of IL-1, IL-6, and IL-13 by mast cells. This alarmin plays a role in inducing pruritus by directly stimulating nerves in psoriasis. IL-33 might trigger Th2 cells to produce IL-4, IL-13, and IL-31.	[20,240,241,243,244,248,249]
IL-1α	Lowered	Conflicting results	IL-1 can induce inflammation by regulating several genes in the skin. Propeptide of IL-1, via CCL-20, causes the development of inflammatory skin lesions and attracts neutrophils to infiltrate the tissue. The propeptide of IL-1 recruits neutrophils and monocytes as an initial step of inflammation IL-1 cytokines can stimulate the production of IL-6, TNF, and IL-33 by mast cells. IL-1 alpha acts synergistically with IL-17A, IL-22, OSM, and TNF alpha in inhibiting the differentiation of keratinocytes. The alarmin stimulates the release of IL-36 from macrophages. IL-1 activates dendritic cells, which release IL-12 and IL-23, leading subsequently to the differentiation of Th1 and Th17 cells.	[18,252,253,254,255,256,260,261]

CCL—chemokine ligand (CC motif); CCR—chemokine receptor; dDCs—dermal dendritic cells; HMGB1—high-mobility group box 1; HSP—heat shock protein; IL—interleukin; TNF—tumor necrosis factor.

**Table 4:** Summary of the impact of selected agents used in the treatment of psoriasis on the expression/concentration of alarmins.

Drug	Mechanism of Action	Impact on Alarmins	References
Etanercept	TNF-α inhibitor	Decreased expression of HMGB1 in the skin. Decreased serum levels of S100A7, S100A12, and S100A8/A9.	[170] [174,176]
Ustekinumab	IL-12/23 inhibitor	Decreased serum levels of S100A7, S100A12, and S100A8/A9. Downregulation of HSP90 in the skin.Increased level of lesional IL-1α in the skin.	[176] [214] [259]
Secukinumab	IL-17a inhibitor	Decreased expression of S10S0A7A, S100A12, and S100A9 in the skin. Decreased serum levels of S100A7, S100A12, and S100A8/A9. Higher serum levels of hBD2. Decreased expression of hBD2 in the skin. Increased level of lesional IL-1α in the skin.	[172,176,183] [183,227,228,259]
Risankizumab	IL-23 inhibitor	Decreased expression of S100A7 in the skin. Decreased expression of hBD2 in the skin.	[184]
Guselkumab	IL-23 inhibitor	Decreased expression of hBD2 in the skin.	[183,227]
Tacrolimus	Calcineurin inhibitor	Inhibition of TNF-α/IL-17A-induced S100A9 expression in the skin.	[185]
Deucravacitinib	TYK2 inhibitor	Decreased expression of S100A9, S100A8, and β-defensin 4A/B.	[186]

hBD—human beta-defensin; HMGB1—high-mobility group box 1; HSP—heat shock protein; IL—interleukin; TNF—tumor necrosis factor.

## Data Availability

Not applicable.
